# A comparison of T1ρ with native T1 and T2 mapping for detecting edema in takotsubo cardiomyopathy

**DOI:** 10.1016/j.jocmr.2025.101965

**Published:** 2025-09-25

**Authors:** Liene Balode, Robert Kelly, David M. Higgins, David Gamble, Dana Dawson, P. James Ross

**Affiliations:** a Aberdeen Biomedical Imaging Centre, University of Aberdeen, Aberdeen, UK; b Aberdeen Cardiovascular and Diabetes Centre, University of Aberdeen, Aberdeen, UK; c Philips, Farnborough, UK

**Keywords:** Quantitative MRI, CMR, Edema, T1ρ mapping, Takotsubo cardiomyopathy

## Abstract

**Background:**

The use of parametric T1 mapping and T2 mapping cardiovascular magnetic resonance (CMR) in takotsubo cardiomyopathy has shown elevated native T1 and T2 relaxation times. In addition to native T1 and T2 mapping, a new native image parametric mapping method using T1 relaxation in the rotating frame (T1ρ) has shown potential to assess myocardial tissue characterization. This study aims to compare T1ρ with native T1 and T2 myocardial mapping in takotsubo cardiomyopathy.

**Methods:**

T1ρ, T2, and native T1 relaxation times were obtained for 51 patients (96% (49/51) female, mean age 69) diagnosed with takotsubo cardiomyopathy and 16 healthy subjects (100% (16/16) female, mean age 41). The baseline scan for the takotsubo cohort was done within 3 weeks after symptom onset, with follow-up scans carried out on average 9 weeks after the baseline scan. Cardiac function and T1ρ, T2, native T1 maps of basal, mid, and apical segments were analyzed.

**Results:**

A significant increase in T1ρ relaxation time was measured in mid and apical segments for the takotsubo baseline cohort compared to takotsubo follow-up cohort (p = 0.0006, p = 0.0011, respectively). A significant increase in T1ρ relaxation time was measured in mid and apical segments for the takotsubo baseline cohort compared to the healthy volunteer cohort (p < 0.0001, p < 0.0001, respectively). Significantly elevated T2 and native T1 relaxation were observed in basal (p = 0.0344, p = 0.0109, respectively), mid (p < 0.0001, p < 0.0001, respectively), and apical (p < 0.0001, p < 0.0001, respectively) segments for takotsubo baseline scans when compared to the takotsubo follow-up cohort. Significant increase in T2 and native T1 relaxation values was also observed in basal (p = 0.0038, p < 0.0001, respectively), mid (p < 0.0001, p < 0.0001, respectively), and apical (p < 0.0001, p < 0.0001, respectively) segments for takotsubo baseline cohort when compared to the healthy volunteer cohort.

**Conclusion:**

In patients with takotsubo cardiomyopathy, T1ρ values were significantly elevated in the mid and apical segments, where edema is more pronounced. In contrast, both T2 and native T1 values were significantly increased across all three segments—basal, mid, and apical. Consequently, native T1 and T2 mapping showed superior ability to detect edema compared to T1ρ mapping.

## Graphical abstract

**Figure:**
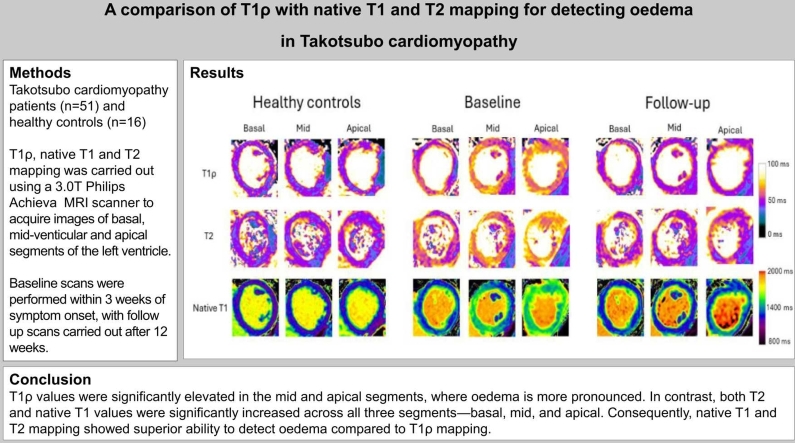

## 1 Background

Takotsubo cardiomyopathy, commonly known as “Broken Heart Syndrome,” is a non-ischemic heart condition that has symptoms such as acute myocardial infarction. Pathogenesis and pathophysiology of takotsubo are not completely known, with catecholamine-induced myocardial injury being the most established and well-known theory [1]. Intense myocardial edema is present in takotsubo syndrome, causing severe left ventricular (LV) dysfunction [1]. Myocardial edema resolves gradually over weeks after the acute phase of takotsubo [1].

Cardiovascular magnetic resonance (CMR) native T1 and T2 mapping are non-invasive methods for assessing and quantifying myocardial tissue composition. Native T1 mapping is often utilized as an indirect marker of myocardial fibrosis, while T2 mapping primarily reflects myocardial edema. Both native T1 and T2 relaxation times are extended due to increased interstitial water content [2], [3].

**Fig. 1:**
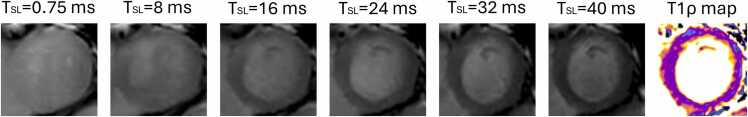
T1ρ-weighted images of the basal segment acquired at different spin-locking durations (T_SL_) and the corresponding T1ρ map

T1ρ tissue distinguishing abilities have prompted investigation of its use in cardiac disease, which has so far focused on fibrosis-dominant cardiomyopathies. T1ρ is an emerging technique that enables endogenous image contrast corresponding to extremely low magnetic fields to be generated using clinical field strength scanners. T1ρ relaxation is sensitive to low-frequency (Hz-kHz) hydrogen motional processes in tissue such as the exchange of protons between water and macromolecules, such as collagen [4]. Several explanations for T1ρ relaxation mechanisms have been proposed, such as dipole-dipole coupling, chemical and exchange, scalar coupling [5]. T1ρ image contrast is generated using a technique known as spin-locking, which sensitizes the magnetic resonance imaging (MRI) experiment to this low-frequency motion. Quantitative T1ρ maps can be obtained by voxel-wise fitting of a series of images with varying T1ρ-weighting (Fig. 1) acquired at different spin-locking durations (T_SL_).

T1ρ imaging has been used in studies of the brain [6], articular cartilage [7], liver [8], muscle [9], and detecting cardiac fibrosis [10]. Recent studies have reported that T1ρ is significantly elevated in patients with cardiac amyloidosis [11], hypertrophic cardiomyopathy [12], and pulmonary hypertension [13] compared with healthy controls. Additionally, increases in T1ρ have been observed in breast cancer patients following anthracycline treatment, suggesting the presence of fibrosis [14].

A study examining T1ρ mapping in both ischemic and non-ischemic cardiomyopathies found that T1ρ values were elevated in the mid-cavity and apical segments of the left ventricle in patients with acute takotsubo cardiomyopathy [15]. By utilizing a larger cohort, this study compares T1ρ with native T1 and T2 mapping in both baseline and follow-up patient populations.

## 2 Methods

### 2.1 Aim

This study aims to compare T1ρ with native T1 and T2 myocardial mapping in takotsubo cardiomyopathy.

### 2.2 Study subjects

Fifty-one patients diagnosed with takotsubo cardiomyopathy were recruited from Aberdeen Royal Infirmary’s acute admissions. The takotsubo cardiomyopathy episode had to have occurred within the past 3 weeks. Patients were diagnosed once fulfillment of Mayo Clinic [16] or European Society of Cardiology [17] criteria had been achieved. The criteria mainly focused on the presence of ballooning of the ventricle (apical, mid, apex, or a combination) demonstrated using CMR. It also focused on the absence of clear-cut coronary vessel involvement, as indicated by angiography imaging. Diagnosis of takotsubo cardiomyopathy was reaffirmed at follow-up, by morphological recovery and hemodynamic improvement. Exclusion criteria included acute or chronic inflammatory disease, concurrent cardiac disease, infection, allergies or contraindications to contrast agents, any contraindications to MRI, and pregnancy. Patient baseline scans were carried out within 3 weeks of symptom onset. Their follow-up scans were carried out on average 9 weeks after the baseline scan. In addition to the takotsubo cohort, a healthy cohort of volunteers was recruited who had no history of cardiovascular disease. Participants were supplied with an information leaflet outlining the purpose of the research. They were allowed at least 24 hours to consider taking part in the study and any questions they had were answered before they provided written informed consent. For those potential participants who were identified with National Health Service Grampian, informed verbal consent was taken over the telephone and written consent was taken at the start of their first study visit at the cardiovascular research facility at the Aberdeen Royal Infirmary if they chose to participate. The study was approved by the North of Scotland Research Ethics Committee and ran in accordance with the Declaration of Helsinki.

### 2.3 Cardiac MR imaging protocol

CMR was carried out using a 3.0T MRI scanner (Achieva dStream, Philips, Best, The Netherlands) at the University of Aberdeen Foresterhill campus. 28 channel receiver coil was used for magnetic resonance (MR) signal reception.

To assess the cardiac function (LV mass, end-diastolic/systolic volumes, and LV ejection fraction), standard cine imaging was performed. A stack of 30 contiguous short-axis images was acquired by retrospectively electrocardiogram (ECG) triggered balanced steady-state free precession (bSSFP) imaging sequence. The bSSFP acquisition sequence parameters: echo time (TE) 1.49 ms, repetition time (TR) 3 ms, field of view (FOV) 250 mm foot − head (FH) × 250 mm anterior to posterior (AP) × 107 mm right to left (RL), voxel size 1.8 mm FH × 1.8 mm AP, 45° flip angle, slice thickness/gap 7/3 mm, acceleration factor 2.9 (Compressed sensitivity encoding [SENSE]).

Early and late gadolinium enhancement (Gadovist, Bayer Heatlthcare, Berlin, Germany, 0.1 mmol/kg) acquired using a spoiled gradient echo inversion recovery sequence: TE 3.0 ms, TR 6.1 ms, FOV 320 mm FH × 356 mm AP × 108 mm RL, voxel size 1.8 mm FH × 2.2 mm AP, 25° flip angle, slice thickness/gap 8/2 mm with swapping of the phase-encoding direction to exclude artifact. Gadolinium was not administered to control and follow-up cohort.

**Fig. 2:**
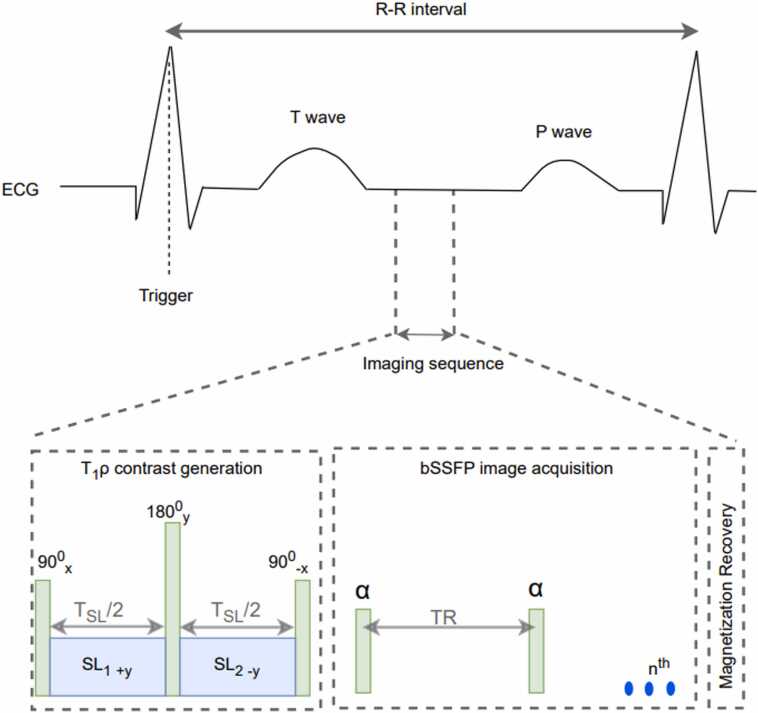
Imaging sequence used to acquire T1ρ-weighted images. Electrocardiogram (ECG) triggering is employed to synchronize the image acquisition with the cardiac cycle. The sequence begins with a magnetization preparation pulse ($90_{x}^{0}-{\mathrm{SL}}_{y}-180_{y}^{0}-{\mathrm{SL}}_{-y}-90_{-x}^{0}$) that starts with a $90^{0}$ pulse along the x-axis, tipping the spins into the transverse plane. A spin-lock (SL) pulse is then applied along the y-axis to “lock” the spins in the transverse plane. This is followed by a $180^{0}$ pulse that refocuses the signal. Another spin-lock pulse is applied along the −y-axis to maintain the spin-locking in the transverse plane, and then a final $90^{0}$ pulse is applied along the −x-axis to return the spins to the longitudinal (z) axis. The magnetization preparation phase is followed by a breath-held single-shot balanced steady-state free precession (bSSFP) image readout, where the flip angle (α) is set to $20^{0}$. The imaging sequence is repeated with different spin-lock times to acquire multiple T1ρ-weighted images. The Specific Absorption Rate of the imaging sequence was 1.26 W/kg. *TR* repetition time

T1ρ imaging was performed using a composite spin-lock pulse (effective field of 500 Hz) comprised of two segments of equal duration but opposite phase to compensate for B1 field inhomogeneities. The segments are separated by a refocusing pulse to reduce sensitivity to B0 field inhomogeneities [18]. Following the spin-lock preparation, a breath-held single-shot bSSFP image readout was used for image acquisition (Fig. 2). T1ρ maps were acquired in three short-axis slices at basal, mid, and apical levels. All T1ρ acquisitions were prospectively triggered to diastole and were acquired during breath hold. The bSSFP acquisition sequence parameters: TR 1.9 ms, FOV 300 mm FH × 300 mm AP × 47 mm RL, voxel size 2 mm FH × 2 mm AP 20° flip angle, slice thickness/gap 10/9 mm, R-R delay 3 beats, acceleration factor 3 (Compressed sensitivity encoding [SENSE]). The imaging sequence was repeated six times with different spin-lock pulse duration (T_SL_): 0.75, 8, 16, 24, 32, and 40 ms.

Native T1 imaging was performed using a balanced gradient echo readout with a Modified Look-Locker Inversion recovery (5s(3s)3s). Sequence parameters: TE 0.94 ms, TR 2.0 ms, FOV 300 mm FH × 300 mm AP × 47 mm RL, voxel size 2 mm FH × 2 mm AP, 20° flip angle, slice thickness/gap 10/8.5 mm, acceleration factor 2 (SENSE).

T2 mapping was performed using gradient- and spin-echo readout. Sequence parameters were TE 0.75 ms, TR 1.92 ms, field of view (FOV) 300 mm feet-head × 300 mm anteroposterior × 48 mm right-left , voxel size 2 mm feet-head × 2 mm anteroposterior, 20° flip angle, and slice thickness/gap 10/9 mm.

### 2.4 CMR image analysis

Once the images were acquired on the scanner, they were transferred to IntelliSpace Portal 11 (Philips Healthcare) for analysis. T1ρ maps were created using a two-parameter fitting model with the signal equation $S\left(T_{\mathrm{sl}}\right)=A*e^{\frac{-T_{\mathrm{sl}}}{T1\rho }}$, where A represents the initial magnetization.

Scans were taken in three regions of the heart: base, mid-cavity, and apex. Scans were obtained in short-axis view, as this offers optimal alignment of the base, mid-cavity, and apex and provides an annular representation of the LV myocardium. Epicardial and endocardial contours were drawn manually, based on the guidelines set out by the Society of Cardiovascular Magnetic Resonance [19]. Segmentation of the myocardial area was also performed manually using the American Heart Association 17-segment model [20]. We excluded the tip of the apex (i.e., segment 17), and therefore, the results were based on averages of 16 segments: 6 for basal, 6 for mid-cavity, and 4 for apical.

The interclass correlation coefficient (ICC) was calculated using SPSS software (IBM Corp., Armonk, New York) for basal, mid, and apical segments by using two-way mixed-effects model with absolute agreement to assess the reliability of Rater 1’s measurements. The ICC was similarly determined for the corresponding T2 and native T1 measurements.

Rater 1 was a PhD student in Medical Sciences whose measurements were used in this report, while Rater 2 was a fifth-year medical student. Both received appropriate training before analyzing the data.

GraphPad Prism 10 software (GraphPad Software, Boston, Massachusetts) was utilized to assess significant differences between baseline and follow-up, as well as between the healthy control and baseline, and healthy control and follow-up groups. P-values were determined using Welch’s t-test (two-tailed) with significance level p < 0.05. Categorical variables in baseline characteristics (Table 1) were analyzed using chi-square test with significance level p < 0.05.

**Table 1:** Baseline characteristics of takotsubo and healthy volunteer cohort.

	Takotsubo patients (n = 51)	Control subject (n = 16)	p value
Female, n (%)	49 (96)	16 (100)	0.421
Male, n (%)	2 (4)	0 (0)	0.421
Age, mean (mean± SD), y	69±8.7	41±11.3	<0.0001
BMI, kg/m^2^ (mean±SD)	26±4.9	25±2.8	0.105
*Medical history, n (%)*			
Hypertension	18 (35)	0 (0)	0.005
Diabetes	5 (10)	0 (0)	0.193
Anxiety	8 (16)	2 (13)	0.755
Depression	8 (16)	1 (6)	0.334
Atrial fibrillation/flutter	7 (14)	0 (0)	0.117
COPD	7 (14)	0 (0)	0.117
Thyroid disease	8 (16)	0 (0)	0.091
*Medication, n (%)*			
ACEi	23 (45)	0 (0)	<0.001
ARB	11 (22)	0 (0)	0.042
MRA	5 (10)	0 (0)	0.193
CCB	4 (8)	0 (0)	0.248
Nitrates	13 (25)	0 (0)	0.024
Antiplatelet	18 (35)	1 (6)	0.025
Anticoagulant	7 (14)	0 (0)	0.117
Diuretic	8 (16)	0 (0)	0.091
Statin	31 (61)	0 (0)	<0.0001
Antidepressants	9 (18)	3 (19)	0.920
Antipsychotics	2 (4)	0 (0)	0.421
*Smoking status (Yes)*			
n (%)	10 (20)	3 (19)	0.940
*Presenting symptom, n (%)*			
Chest pain	41 (80)	0 (0)	<0.0001
Breathlessness	7 (14)	0 (0)	0.117
Syncope/collapse	1 (2)	0 (0)	0.573
Abdominal pain	1 (2)	0 (0)	0.573
Diarrhea	1 (2)	0 (0)	0.573
*Presenting ECG, n (%)*			
ST-segment elevation	17 (34)	-	-
LBBB	2 (4)	-	-
TWI	30 (59)	-	-
No changes	2 (4)	-	-
*Angiography, n (%)*			
No angiography	9 (18)	-	-
Normal	22 (43)	-	-
Mild or severe plaque	20 (39)	-	-
*Stressor*			
Physical	13 (25)	-	-
Emotional	20 (39)	-	-
None	18 (35)	-	-
*Baseline blood levels (average)*			
BNP	164	-	-
Troponin	1912	-	-

*BMI* body mass index, *COPD* chronic obstructive pulmonary disease, *ACEi* angiotensin-converting enzyme inhibitors, *ARB* angiotensin receptor blockers, *MRA* mineralocorticoid receptor antagonists, *CCB* calcium channel blockers, *LBBB* left bundle branch block, *BNP* brain natriuretic peptide, *ECG* electrocardiogram, *SD* standard deviation, *TWI* T wave inversion

The cine-CMR and LGE images were evaluated by consultant cardiologists at Aberdeen Royal Infirmary.

## 3 Results

### 3.1 Study subjects

In total, we included 51 patients diagnosed with takotsubo cardiomyopathy and 16 healthy volunteers. Baseline characteristics of each cohort are displayed in Table 1.

The mean age of the 51 patients was 69 (range: 40–83), whereas the mean age of the controls was 41 (range: 23–53), a difference that is statistically significant (P < 0.0001). For the takotsubo cohort, 49 were female (96%) which matched our healthy cohort, whom were all female. Comorbidities were significantly more prevalent in the takotsubo patients, reflected by the number of medications they were taking (Table 1).

### 3.2 Cardiac function measurements

Baseline scans predominantly showed wall motion abnormalities (WMA) in the mid anterior, mid anteroseptal, mid inferoseptal, apical anterior, and apical septal regions (Supplementary Table 1).

Measurements of LV size and function were taken for takotsubo and healthy volunteer cohorts, and corresponding p values were calculated (Table 2).

**Table 2:** Left ventricular (LV) characteristics of takotsubo and healthy volunteer cohort.

Measurement	Baseline	Follow-up	Controls	p value Baseline vs controls	p value Follow-up vs controls	p value Baseline vs follow-up
LV mass (g/m^2^) average	71.0±13.9 (n = 50)	60.5±12.8 (n = 40)	68.0±8.3 (n = 14)	0.3204	0.0172*	0.0003**
LV end-diastolic volume (mL) average	69.0±12.0 (n = 50)	69.0±13.0 (n = 40)	63.5±13.6 (n = 14)	0.1835	0.1978	0.9985
LV end-systolic volume (mL) average	27.3±11.1 (n = 50)	23.9±8.7 (n = 40)	20.9±4.4 (n = 14)	0.0022***	0.1111	0.1078
LV ejection fraction (%) average	62.4±12.9 (n = 50)	66.2±8.0 (n = 40)	66.5±4.4 (n = 14)	0.0604	0.8400	0.0906

*SD* standard deviation, *LV* left ventricular

The values are displayed as mean ± SD. Not significant (ns) P > 0.05

* P ≤ 0.05

** P ≤ 0.001

^***^ P ≤ 0.01 ^****^ P ≤ 0.0001

### 3.3 LGE, T1ρ, T2, native T1 measurements

Gadolinium was administered only for the baseline scans and no LGE was observed in any patient.

For T1ρ mapping, 22 segments were excluded due to artifacts and a further 50 segments were not obtained due to subjects being lost at follow-up. For T2 mapping, 62 segments were excluded due to image artifacts and 27 segments were not obtained due to subjects being lost at follow-up. For native T1 mapping, 10 segments were excluded due to image artifacts and 42 segments were not obtained due to subjects being lost at follow-up. Table 3 provides details on the sample size for each segment across the different cohorts’ T1ρ/ T2/native T1 scans. The Supplementary file contains examples of segments that were excluded due to image artifacts (Supplementary Fig. 1).

**Table 3:** Measured T1ρ, T2, and native T1 relaxation.

Segment	Relaxation (ms)	Baseline	Follow-up	Controls	p value Baseline vs controls	p value Follow-up vs controls	p value Baseline vs follow-up
Basal	T1ρ	47.8±2.9 (n = 46)	47.0±3.8 (n = 41)	47.1±3.9 (n = 11)	0.5703	0.9610	0.2780
	T2	50.5±3.9 (n = 39)	48.7±3.3 (n = 35)	46.3±3.6 (n = 11)	0.0038*	0.0695	0.0344**
	Native T1	1305±62.3 (n = 46)	1272±56.2 (n = 42)	1236±24.6 (n = 13)	<0.0001***	0.0024*	0.0109**
Mid	T1ρ	49.8±4.0 (n = 43)	46.6±4.2 (n = 39)	44.3±2.8 (n = 15)	<0.0001***	0.0268*	0.0006***
	T2	54.5±5.9 (n = 42)	47.9±3.0 (n = 37)	44.9±2.9 (n = 10)	<0.0001***	0.0109*	<0.0001***
	Native T1	1358±80.1 (n = 47)	1264±39.2 (n = 41)	1236±28.9 (n = 13)	<0.0001***	0.0090*	<0.0001***
Apical	T1ρ	51.9±5.6 (n = 39)	48.0±4.7 (n = 38)	44.1±3.1 (n = 10)	<0.0001***	0.0053	0.0011*
	T2	57.7±7.2 (n = 42)	49.3±3.1 (n = 37)	46.6±1.7 (n = 12)	<0.0001***	0.0004****	<0.0001***
	Native T1	1401±81.3 (n = 46)	1290±57.1 (n = 41)	1270 ±35.9 (n = 13)	<0.0001***	0.1589	<0.0001***

*SD standard deviation*

Although 51 takotsubo patients and 16 healthy controls were initially recruited for the study, the actual sample size (n) for each segment was reduced due to image artifacts or lost follow-up. The values are displayed as mean ± SD of basal, mid, and apical segments. Not significant (ns) p > 0.05

* p ≤ 0.01

** p ≤ 0.05

*** p ≤ 0.0001

**** p ≤ 0.001

**Fig. 3:**
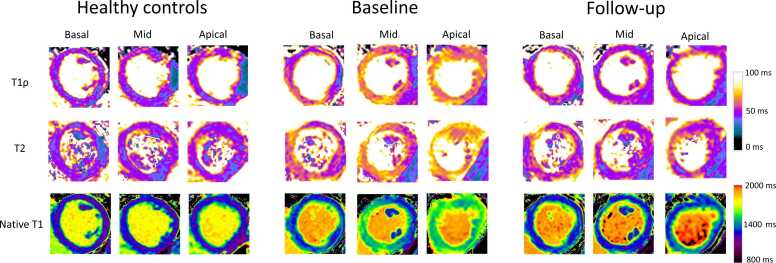
Quantitative T1ρ, T2, and native T1 maps of basal, mid, and apical segments. Maps are acquired from a healthy volunteer and patient of takotsubo baseline and follow-up cohorts. For T1ρ, a banding artifact can be observed in healthy controls which is most likely induced by field-inhomogeneity effects during the spin-locking pulse [18]. For T2 mapping, a banding artifact is also observed in healthy controls. Regions affected by the banding artifact were excluded from the segment analysis

Fig. 3 shows quantitative T1ρ, T2, and native T1 maps acquired from a patient of takotsubo cohort and a healthy volunteer. The obtained T1ρ, T2, and native T1 values from all the scans are displayed in Table 3.

**Fig. 4:**
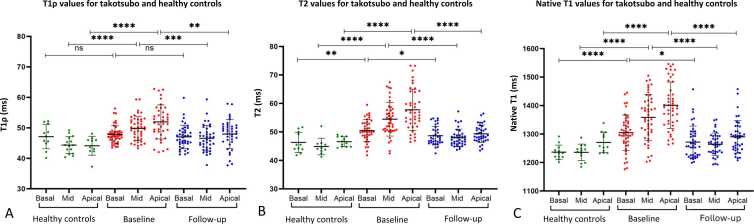
T1ρ (A), T2 (B), and native T1 (C) relaxation measurements of basal, mid, and apical segments from takotsubo and healthy volunteer cohorts. Not significant (ns) p > 0.05, *p ≤ 0.05, **p ≤ 0.01, ***p ≤ 0.001, ****p ≤ 0.0001.

A significant increase in T1ρ relaxation time was measured in mid and apical segments for the takotsubo baseline cohort compared to takotsubo follow-up cohort (p = 0.0006, p = 0.0011, respectively). A significant increase in relaxation time was measured in mid and apical segments for the takotsubo baseline cohort compared to the healthy volunteer cohort (p < 0.0001, p < 0.0001, respectively). Basal segments showed non-significant changes in T1ρ relaxation between baseline cohort and follow-up cohort (p = 0.2780) and between baseline cohort and the healthy volunteer cohort (p = 0.5703) (Fig. 4A).

Significantly elevated T2 and native T1 relaxation were observed in basal (p = 0.0344, p = 0.0109, respectively) mid (p < 0.0001, p < 0.0001, respectively), and apical (p < 0.0001, p < 0.0001, respectively) segments for takotsubo baseline scans when compared to the takotsubo follow-up cohort.

Significant increase in T2 and native T1 relaxation values were also observed in basal (p = 0.0038, p < 0.0001, respectively), mid (p < 0.0001, p < 0.0001, respectively), and apical (p < 0.0001, p < 0.0001, respectively) segments for takotsubo baseline cohort when compared to the healthy volunteer cohort (Fig. 4B, C).

When evaluating each of the 16 segments individually (Supplementary Table 1), it can be observed that T1ρ, T2, and native T1 exhibit the largest percentage decrease between the follow-up and baseline in the mid anterior (7.7%, 13.2%, and 8.4%, respectively), mid anteroseptal (9.3%, 16.3%, and 7.9%, respectively), mid inferoseptal (8.3%, 13.1%, and 7.4%, respectively), apical anterior (7.6%, 15.4%, and 8.3%, respectively), and apical septal (11.7%, 17.9%, and 9.3%, respectively) segments.

ICC for T1ρ, T2, and native T1 values were 0.92 (95% confidence interval [CI]: 0.40-0.98), 0.84 (95%CI: 0.45-0.94), 0.95 (95%CI: 0.44-0.98) for basal, 0.93 (95%CI: 0.62-0.98), 0.84 (95%CI: 0.56-0.93), 0.95 (95%CI: 0.63-0.99) for mid and 0.96 (95%CI: 0.47-0.99), 0.88 (95%CI: 0.40-0.97), 0.98 (95%CI: 0.57-0.99) for apical segments, respectively.

## 4 Discussion

Objective markers such as normal cardiac morphology and LV ejection fraction are typically used to gauge recovery in takotsubo patients. While these markers often return to normal relatively quickly, many patients still experience symptoms and a reduced quality of life, suggesting a more complex disease progression [1].

LV mass was shown to be greatest in the baseline takotsubo patients (Table 2) and this is in keeping with the morphological ballooning of the apex and edematous, inflammatory response. Furthermore, takotsubo follow-up showed a very significant reduction in LV mass, compared to baseline values, suggesting that the acute phase edematous changes had resolved.

This study found that T1ρ, T2, and native T1 values were significantly elevated in the acute phase of takotsubo and significantly reduced at follow-up scan to values observed in a healthy cohort. This could be explained by the increased free water content due to myocardial edema which resolves gradually over weeks after the acute phase of takotsubo.

T1ρ, T2, and native T1 mapping showed basal-to-apical gradient of increasing T1ρ/T2/native T1 values at baseline. T1ρ mapping revealed significant changes in T1ρ relaxation time for the mid and apical segments, with non-significant changes observed in the basal segment but T2 and native T1 values were significantly elevated across all three segments when comparing healthy controls to baseline and follow-up to baseline. The notable increase in T2 in the basal segment may be attributed to the fact that T2 is specific and sensitive to edema [21] and quickly loses sensitivity after the acute phase. The significant increase in native T1 value for basal segment in acute phase is also likely attributed to edema presence.

WMA in baseline scans were predominantly observed in the mid anterior, mid anteroseptal, mid inferoseptal, apical anterior, and apical septal regions. These areas exhibited the most significant increase in T1ρ values when compared to follow-up. Similarly, T2 and native T1 values showed the largest increase in these regions, with notable increases across all six mid and four apical segments. It has been reported that edema is not only confined to regions of abnormal contractility but is present to a lesser extent within the entirety of the ventricular myocardium in takotsubo [1]. The results suggest that T2 and native T1 mapping exhibit greater detection capability for edema compared to T1ρ mapping.

T1ρ has emerged as a promising technique for detecting cardiac fibrosis based on the presence of protein molecules directly, rather than indirectly through their effects on water, which is the case for T1 and T2 relaxation [4]. Schwarz et al. reported that native T1 and extracellular volume fraction (ECV) remained significantly elevated in Takotsubo patients at both baseline and 4-month follow-up, suggesting that diffuse fibrosis could develop as early as 4 months in more severe cases of takotsubo [22]. While we cannot completely exclude the possibility that the elevated T1ρ values observed at the 12-week follow-up are due to fibrosis, this is unlikely. Both T1ρ and T2 values remain slightly elevated compared to the healthy cohort, which more strongly suggests the presence of residual edema rather than fibrotic changes. Furthermore, no cases were observed in which T1ρ remained elevated at follow-up while T2 and native T1 had returned to normal levels seen in the healthy cohort.

Further studies are needed to extend follow-up imaging in takotsubo patients beyond 12 weeks, to investigate whether T1ρ mapping can detect longer-term cellular changes. Specifically, this could help determine if persistent elevations in T1ρ, LGE, and/or ECV along with increased native T1—suggestive of focal or diffuse fibrosis [23]—are present even as T2 values normalize, indicating resolution of edema.

When comparing the healthy control T1ρ values from this study with those reported in recent cardiac imaging applications of T1ρ, we observed notable variations both relative to our findings and among the recent studies. These discrepancies may be attributed to differences in spin-lock pulse preparation parameters, such as T_SL_, as well as variations in B1 and B0 field strengths across studies.

In this study, a spin-locking field of 500 Hz with spin-lock times of [0.75, 8, 16, 24, 32, 40 ms] was applied. Thompson et al. and Wang et al. used a combination of 400 and 500 Hz spin-locking fields with spin-lock times of [0, 2, 10, 18, 26, 34, 42, 50 ms] [12], [13]. Deng et al., Wang et al., and Li et al. reported B1 field strengths of 400, 298, and 300 Hz with corresponding spin-lock times of [0, 10, 20, 30 ms], [30, 44, 54 ms], and [1.2, 13, 27, 40 ms], respectively [11], [14], [24]. These variations highlight the need for careful standardization to establish reliable T1ρ reference values for future studies.

## 5 Limitations

The baseline scans were performed within 3 weeks of symptom onset. Due to the transient nature of the acute phase of takotsubo cardiomyopathy, it is possible that some of our patients experienced partial or even full resolution of the edematous changes and apical ballooning by the time of their baseline scan. Medina et al. demonstrated that LV function often resolves within 3 weeks, suggesting that baseline scans should ideally be conducted much sooner after symptom onset [25]. If our scans had been conducted within 48 h, when LV systolic dysfunction is typically most pronounced, the results might have shown more significant differences between the baseline and follow-up scans.

The mean age of the healthy volunteer cohort is lower (average age 41 years) than the mean age of takotsubo cohort (average age 69 years) and it is known that T1 relaxation time is slightly lower with an increasing age [26]. The effect of age on myocardial T1ρ values has not been widely explored. An average age difference of 28 years may influence other important factors, such as comorbidities. A recent study examined the dependence of T1ρ values on gender and age and reported a significant difference across age decades in healthy male cohorts. In contrast, no significant age-related differences were observed among healthy female cohorts [24]. Since both the takotsubo cardiomyopathy and healthy control groups in our study consisted predominantly of female participants, our findings are therefore limited to the female population.

The reproducibility and replicability of T1ρ values for the healthy control group were not assessed. As a result, it is challenging to evaluate the precision of the T1ρ technique in healthy subjects. Furthermore, there are no established reference values for T1ρ in normal myocardial tissue, as T1ρ values can vary depending on different system vendors, field strengths, and pulse sequences [4].

Extended follow-up scans beyond 3 months reveal cellular changes, such as microscopic fibrosis, highlighting the long-term implications of takotsubo [22]. Whether T1ρ values can detect this permanent cellular change remains to be explored.

Lack of histological evidence evaluating T1ρ in takotsubo make it challenging to give definitive conclusions about the underlying pathophysiology. Histology would allow for better understanding of T1ρ ability to detect microstructural changes in myocardial tissue.

Twenty-two segments out of 304 were excluded due to banding artifacts for T1ρ mapping. Using adiabatic spin-lock pulses instead of continuous wave spin-lock pulses would potentially be a better option as adiabatic pulses are more robust against field inhomogeneities compared to conventional continuous wave spin-lock pulses [18].

## 6 Conclusion

The results suggest that T2 and native T1 mapping are superior to identify edema in takotsubo cardiomyopathy compared to T1ρ mapping. Further research is needed to determine whether T1ρ MRI can detect long-term cellular changes, such as microscopic fibrosis, in Takotsubo cardiomyopathy.

## Funding

This study is funded by the 10.13039/501100000274British Heart Foundation (PG/18/35/33786).

## Author contributions

**David Gamble:** Writing – review & editing, Resources, Project administration, Investigation. **Dana Dawson:** Writing – review & editing, Supervision, Resources, Funding acquisition, Conceptualization. **David M. Higgins:** Software. **P. James Ross:** Writing – review & editing, Supervision, Project administration, Methodology, Investigation. **Liene Balode:** Writing – original draft, Visualization, Software, Formal analysis. **Robert Kelly:** Writing – original draft, Formal analysis.

## Ethics approval and consent

All study subjects provided written informed consent. Participant recruitment was approved by the North of Scotland Research Ethics Committee (19/NS/0020).

## Consent for publication

All healthy subjects and patients gave informed consent for publication.

## Declaration of competing interests

The authors declare that they have no known competing financial interests or personal relationships that could have appeared to influence the work reported in this paper.

## Data Availability

The datasets used and analyzed during the current study are available from the corresponding author on reasonable request.

## Footnotes

Appendix A Supplementary data associated with this article can be found in the online version at doi:10.1016/j.jocmr.2025.101965.
