# Supplementary material for: A comparison of T1ρ with native T1 and T2 mapping for detecting edema in takotsubo cardiomyopathy
Source: J Cardiovasc Magn Reson. 2025 Sep 25;27(2):101965. doi: 10.1016/j.jocmr.2025.101965 (PMC12766611; doi:10.1016/j.jocmr.2025.101965)
Supplement: Supplementary file 1 — Supplementary material [file mmc1.docx]

| **Segment** | | **WMA**  **(%)** | **T1ρ** | | | | **T2** | | | | **Native T1** | | | |
| --- | --- | --- | --- | --- | --- | --- | --- | --- | --- | --- | --- | --- | --- | --- |
|  |  |  | Baseline  (ms) | Follow-up (ms) | P value | Percentage change (%) | Baseline | Follow-up | P value | Percentage change (%) | Baseline | Follow-up | P value | Percentage change (%) |
| **Basal** | anterior | 0.6 | 48.0 ± 4.1 | 45.5 ± 4.4 | 0.0130* | 5.2 | 51.9 ± 6.0 | 49.3 ± 5.5 | 0.0860 | 5.0 | 1315 ± 87.3 | 1266 ± 67.6 | 0.0045** | 3.7 |
|  | anteroseptal | 2.8 | 48.1 ± 4.1 | 47.6 ± 4.2 | 0.5587 | 1.1 | 51.6 ± 5.4 | 49.6 ± 6.3 | 0.1751 | 3.9 | 1320 ± 73.1 | 1281 ± 67.9 | 0.0130* | 2.9 |
|  | inferoseptal | 3.3 | 47.3 ± 4.4 | 46.4 ± 4.2 | 0.3677 | 1.8 | 50.2 ± 5.5 | 48.3 ± 4.3 | 0.1043 | 3.8 | 1294 ± 68.8 | 1263 ± 49.0 | 0.0179* | 2.4 |
|  | inferior | 1.7 | 48.6 ± 5.9 | 47.9 ± 4.3 | 0.6010 | 1.3 | 50.0 ± 4.7 | 50.6 ± 4.9 | 0.5981 | -1.2 | 1301 ± 65.6 | 1290 ± 82.6 | 0.4965 | 0.8 |
|  | inferolateral | 0.5 | 46.9 ± 4.1 | 46.6 ± 5.3 | 0.8106 | 0.6 | 49.5 ± 5.9 | 47.9 ± 4.9 | 0.2051 | 3.3 | 1298 ± 86 | 1279 ± 70.6 | 0.2536 | 1.5 |
|  | anterolateral | 0.5 | 48.6 ± 4.7 | 46.6 ± 5.4 | 0.0810 | 4.2 | 49.7 ± 6.6 | 46.4 ± 3.8 | 0.0124* | 6.6 | 1292 ± 89.4 | 1253 ± 58.5 | 0.0180* | 3.0 |
| **Mid** | anterior | 10.5 | 50.5 ± 6.0 | 46.7 ± 6.1 | 0.0071** | 7.7 | 57.0 ± 8.1 | 49.3 ± 5.2 | <0.0001**** | 13.2 | 1375 ± 95.7 | 1260 ± 49.0 | <0.0001**** | 8.4 |
|  | anteroseptal | 12.2 | 51.2 ± 5.5 | 46.5 ± 5.5 | 0.0003*** | 9.3 | 57.0 ± 8.7 | 47.7 ± 3.3 | <0.0001**** | 16.3 | 1383 ± 102.1 | 1273 ± 51.6 | <0.0001**** | 7.9 |
|  | inferoseptal | 10.5 | 50.6 ± 5.8 | 46.4 ± 4.5 | 0.0008*** | 8.3 | 54.5 ± 8.0 | 47.3 ± 3.7 | <0.0001**** | 13.1 | 1365 ± 98.3 | 1263 ± 44.4 | <0.0001**** | 7.4 |
|  | inferior | 6.6 | 48.6 ± 6.1 | 47.9 ± 4.8 | 0.5914 | 1.5 | 53.5 ± 7.0 | 47.1 ± 3.2 | <0.0001**** | 11.9 | 1339 ± 88.2 | 1269 ± 57.1 | <0.0001**** | 5.2 |
|  | inferolateral | 4.4 | 47.6 ± 5.0 | 45.7 ± 6.5 | 0.1771 | 4.1 | 52.4 ± 7.1 | 47.8 ± 4.6 | 0.0025** | 8.7 | 1333 ± 92.6 | 1255 ± 52.1 | <0.0001**** | 5.8 |
|  | anterolateral | 5.0 | 49.6 ± 4.9 | 46.8 ± 5.5 | 0.0214* | 5.7 | 52.2 ± 6.8 | 47.6 ± 4.5 | 0.0008*** | 8.8 | 1348 ± 93.7 | 1268 ± 54.3 | <0.0001**** | 5.9 |
| **Apical** | anterior | 11.6 | 53.0 ± 7.5 | 49.0 ± 5.8 | 0.0129* | 7.6 | 59.3 ± 8.2 | 50.2 ± 6.3 | <0.0001**** | 15.4 | 1409 ± 90.2 | 1292 ± 75.4 | <0.0001**** | 8.3 |
|  | septal | 13.8 | 53.1 ± 6.7 | 46.8 ± 5.2 | <0.0001**** | 11.7 | 59.2 ± 8.3 | 48.6 ± 4.4 | <0.0001**** | 17.9 | 1412 ± 93.0 | 1281 ± 64.4 | <0.0001**** | 9.3 |
|  | inferior | 8.3 | 49.8 ± 6.0 | 48.1 ± 6.9 | 0.3104 | 3.4 | 56.3 ± 7.6 | 49.5 ± 3.4 | <0.0001**** | 11.9 | 1391 ± 96.1 | 1283 ± 61.7 | <0.0001**** | 7.8 |
|  | lateral | 7.7 | 50.4 ± 5.6 | 48.5 ± 6.1 | 0.1968 | 3.8 | 56.0 ± 7.8 | 48.9 ± 3.1 | <0.0001**** | 12.6 | 1393 ± 84.1 | 1304 ± 65.5 | <0.0001**** | 6.4 |

Supplementary Table 1. Measured T1ρ, T2 and native T1 relaxation of each of the 16 segments. The wall motion abnormality (WMA) per segment is calculated as a percentage of the total abnormalities reported at baseline. The percentage change reflects the difference in relaxation times between the baseline and follow-up measurements. The values are displayed as mean ± SD of basal, mid and apical segment. Not significant (ns) P > 0.05, * P ≤ 0.05, ** P ≤ 0.01, *** P ≤ 0.001, **** P ≤ 0.0001.


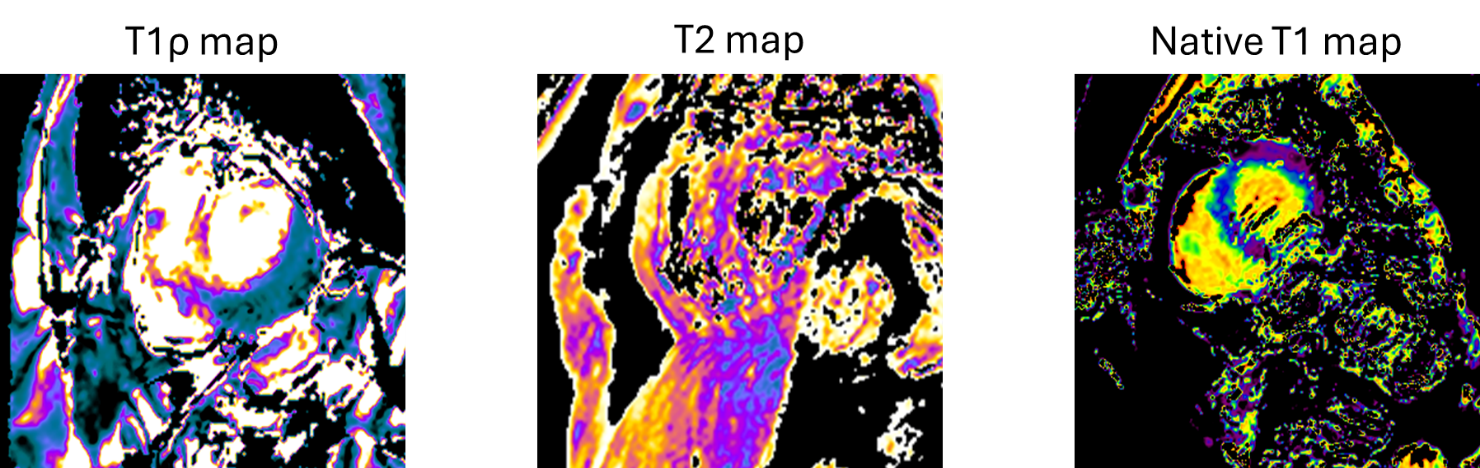


Supplementary Figure 1. Examples of segments excluded due to image artifacts. The T1ρ map is affected by banding artifacts likely induced by field inhomogeneity effects during the spin-locking pulse. The T2 map is likely affected by patient motion.
